# Systematic Identification, Fragmentation Pattern, And Metabolic Pathways of Hyperoside in Rat Plasma, Urine, And Feces by UPLC-Q-Exactive Orbitrap MS

**DOI:** 10.1155/2022/2623018

**Published:** 2022-09-13

**Authors:** Li Ji, Wenjun Shi, Yanling Li, Jing He, Guang Xu, Ming Qin, Yuying Guo, Qun Ma

**Affiliations:** School of Chinese Materia Medica, Beijing University of Chinese Medicine, Beijing 102488, China

## Abstract

Hyperoside is a natural flavonol glycoside, which has antioxidation, antitumor, and anticancer activities together with other healthy effects like improving cardiovascular function, protecting the liver, and regulating the immune system. It is a popular compound used in the traditional Chinese medicine and different studies on hyperoside are present in the literature. However, studies on the metabolism of hyperoside *in vivo* were not comprehensive. In this study, UPLC-Q-Exactive Orbitrap MS technology was used to establish a rapid and comprehensive analysis strategy to explore the metabolites and metabolic process of hyperoside in rats. The metabolites of hyperoside were systematically identified in rat plasma, urine, and feces. According to the hyperoside standard substance and relevant works of literature, a total of 33 metabolites were identified, including 16 in plasma, 31 in urine, and 14 in feces. Among them, the metabolites quercetin and dihydroquercetin were unambiguously confirmed by comparison with standard substances. In addition, 13 metabolites had not been reported in hyperoside metabolism-related articles at present. The metabolic reactions of hyperoside *in vivo* were further explored, including phase I metabolism (hydroxylation, dehydroxylation, glycoside hydrolysis, hydrogenation, and hydration) and phase II metabolism (methylation, acetylation, sulfation, and glucuronide conjugation). The fragment ions of hyperoside and its metabolites were usually produced by glucoside bond hydrolysis, the neutral loss of (CO + OH), COH, CO, O, and Retro-Diels Alder (RDA) cleavage. In conclusion, this study comprehensively characterized the metabolism of hyperoside in rats, providing a basis for exploring its various biological activities.

## 1. Introduction

**Figure 1:**
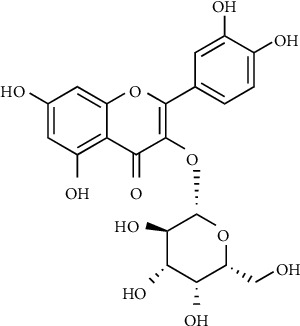
The structure of hyperoside.

Hyperoside, also known as quercetin-3-O-*β*-D-galactoside, is a natural flavonol glycoside and is widely distributed in *Crataegus azarolus* L [1], *Lonicera japonica* Thunb [2], *Ligularia fischeri* (Ledeb.) Turcz [3], and so on. The structure of hyperoside is shown in Figure 1. Modern pharmacological studies have found that hyperoside possesses a broad spectrum of biological activities, including anti-inflammatory [4], antioxidation [5], hypoglycemic [6], antidepressant [7], antitumor [8], and anticancer [9] together with other healthy effects like improving cardiovascular function [10], protecting the liver [11], and regulating the immune system [12].

In view of many biological activities and pharmacological effects, hyperoside has been widely studied [13]. As the basis for research, studies on its metabolites *in vivo* have been reported. For example, Guo et al. [14] used UPLC-Q-TOF-MS to characterize 12 different types of hyperoside metabolites (including the prototype) in rat plasma, urine, and bile. Yang et al. [15] studied the interaction between hyperoside and human intestinal bacteria and detected the prototype compound and six other metabolites from the isolated bacterial samples. However, the metabolites analysis and metabolic process of hyperoside *in vivo* are not comprehensive at present. Therefore, it is necessary to study the metabolites of hyperoside systematically in rat plasma, urine, and feces, so as to deeply make a further comprehensive characterization of hyperoside in rats and explore its pharmacological mechanism.

At present, liquid chromatography-mass spectrometry (LC-MS) and gas chromatography-mass spectrometry (GC-MS) are commonly used in metabolism analysis *in vivo* [16]. UPLC-Q-Exactive Orbitrap MS has the advantages of high resolution, high-quality accuracy, high sensitivity, and wide scanning range compared with other detection instruments [17]. It can realize efficient and rapid identification and analysis of compounds. For example, San Miao Wan (SMW) is a prescription that consists of Phellodendri Chinensis Cortex, Atractylodis Lanceae Rhizoma, and Achyranthis Bidentatae Radix and has a complex composition. Ma et al. [18] used UPLC-Q-Exactive Orbitrap MS to characterize 76 constituents of SMW and to detect 113 metabolites in rat plasma. Therefore, in this study, UPLC-Q-Exactive Orbitrap MS was used to systematically analyze hyperoside and its metabolites in rats after an oral administration, which would help to find potential active metabolites of hyperoside.

This study aimed to use UPLC-Q-Exactive Orbitrap MS to comprehensively and accurately analyze hyperoside and its metabolites in rats after an oral administration. The metabolites of hyperoside in rat plasma, urine, and feces were identified systematically, the fragmentation patterns were summarized, and the metabolic pathways of hyperoside were further explored, which would help to find the target of active components, promote the efficient development and utilization of hyperoside.

## 2. Materials and Methods

### 2.1. Chemicals and Reagents

Hyperoside, quercetin, dihydroquercetin (all with a purity of >98%), and carboxymethylcellulose sodium salt (CMC-Na) were purchased from Shanghai Yuanye Biotechnology Co. Ltd. Acetonitrile (Fisher, USA), methanol (Fisher, USA), and formic acid (Fisher, USA) were LC-MS grade. Purified water was obtained from Watsons Water (Watsons, CHN).

### 2.2. Animal Experiments

Ten healthy Sprague-Dawley rats, (male, SPF grade), weighing 200 ± 20 g, were purchased from Beijing huafukang Biotechnology Co., Ltd. (Beijing), license number: SCXK (jing) 2019-0008. All rats were kept in an animal room with darkness and light alternated for 12 hours. The room temperature was 23 ± 2°C and the relative humidity was 60 ± 5%. Free access to food and water. After keeping the adaptation period for 3 days, the rats were randomly divided into two groups: group A, the plasma group; group B, the urine and fecal group. Before the experiment, they fasted for 12 hours but drank freely. All experiments were carried out in accordance with the approved Regulations on the Management of Experimental Animals and the Detailed Rules on the Management of Chinese Medical Experimental Animals. All animal experiments were approved by the Medical Ethics Review Committee of Animal Experiments of Beijing University of Chinese Medicine (NO: BUCM-4-2022040602-2003).

Hyperoside was dissolved in 0.1% carboxymethyl cellulose sodium salt (CMC-Na) buffer solution up to 50 mg/mL and given to rats at a dose of 350 mg/kg. In group A, the plasma samples were collected from the orbital venous plexus of rats at 10 min, 30 min, 1 h, 1.5 h, 2 h, 4 h, and 6 h after administration, respectively. The obtained plasma samples were centrifuged at 3500 rpm, 4°C for 10 min, and the supernatant was taken. Rats in group B were placed in metabolic cages after administration, and the urine and feces were collected in time periods: 0–4 h, 4–8 h, 8–12 h, and 12–24 h. The collected urine samples were centrifuged at 12000 rpm in a 4°C high-speed freezing centrifuge for 15 min, and then the supernatant was taken. The fecal samples were dried in the fume hood, then crushed and sealed. The plasma of group A and the urine and feces of group B collected before administration were as blank samples. All the above-given samples were stored at −80°C for later use.

### 2.3. Biological Samples Preparation

#### 2.3.1. Preparation of Plasma Samples

50 *μ*L of plasma was taken from different blood collection points of each rat from group A and combined. 5.25 mL of acetonitrile was added to precipitate the proteins, followed by 5 min of a vortex and a centrifugation step at 12000 rpm for 15 min at 4°C. The supernatants were collected and dried under a nitrogen stream.

#### 2.3.2. Preparation of Urine Samples

20 *μ*L urine of each rat from group B was collected at 4 time periods: 0–4 h, 4–8 h, 8–12 h, and 12–24 h and mixed evenly. 1.2 mL of acetonitrile was added to precipitate the proteins, followed by 5 min of a vortex and a centrifugation step at 12000 rpm for 15 min at 4°C. The supernatants were collected and dried under a nitrogen stream.

#### 2.3.3. Pretreatment of Feces Samples

20 mg feces of each rat from group B at 4 time periods: 0–4 h, 4–8 h, 8–12 h, and 12–24 h, were mixed evenly and 4 mL of acetonitrile was added. After 5 min of a vortex, the ice bath ultrasound was performed for 60 min, followed by a centrifugation step at 12000 rpm for 15 min at 4°C. The supernatants were collected and dried under a nitrogen stream.

Before analysis, all pretreated samples were dried under nitrogen, and the residue was redissolved with 200 *μ*L 10% methanol, followed by 5 min of a vortex, centrifugation at 12000 rpm for 15 min at 4°C, and then the supernatant was taken. Finally, the supernatant of each sample was injected into UPLC-Q-Exactive Orbitrap MS for analysis.

### 2.4. Instruments and Conditions

Chromatographic separation was performed using an ACQUITY UPLC BEH C_18_ column (2.1 × 100 mm, 1.7 *μ*m; Waters Corp., USA) with a temperature of 20°C. The mobile phase consisted of water with 0.1% (volume fraction) formic acid (A) and acetonitrile (B). The gradient elution procedure was 0–8 min 10–80% B; 8–11 min, 80–100% B; 11–13 min 100% -10% B; 13–15 min 10% B at a rate of 0.2 mL/min. The sample chamber temperature was 10°C. The injection volume was 2 *μ*L.

For mass spectrum acquisition, a Q-Exactive Orbitrap MS (Thermo Fisher, USA) was used with electrospray ionization source (ESI) in negative mode and MS conditions were optimized as follows: sheath gas flow rate was 40 arb and aux gas flow rate was 10 arb; spray voltage was at 3.0 kV; capillary temperature was at 350°C; high-resolution MS scanning was performed under full scan/dd-MS^2^ covered the range from *m/z* 100 to 1200 with a full scan at a resolution of 70000 and dd-MS^2^ resolution of 17500.

### 2.5. Data Processing and Analysis

The chromatograms were imported into Xcalibur 3.0 software to calculate the mass to charge ratio of possible excimer ions of the compound. Based on this, data processing such as peak extraction and peak matching were performed on the collected spectrograms. Based on the retention time and characteristic fragment ions of standards, the compounds were quickly annotated. The error was less than 10 ppm.

## 3. Results and Discussion

**Figure 2:**
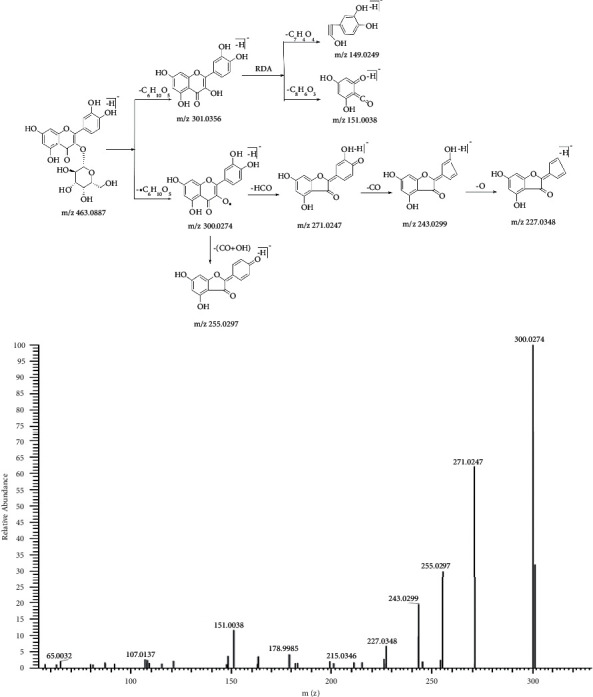
The fragment ion spectra and proposed fragmentation pathways of hyperoside standard.

Hyperoside is a flavonol glycoside and has multiple phenolic hydroxyl groups. So it tends to lose H and has a negative charge when ionized. The fragment ion spectra and proposed fragmentation pathways of hyperoside in negative mode are shown in Figure 2. The [M-H]^−^ ion at *m/z* 463.0887 lost galactoside and galactoside radical, respectively, to generate aglycone ion at *m/z* 301.0356 and aglycone radical ion at *m/z* 300.0274. The ion at *m/z* 300.0274 further lost (CO + OH) to generate the product ion at *m/z* 255.0297. Other product ions at *m/z* 271.0247, 243.0299, and 227.0348 were also generated after a consecutive loss of COH, CO, and O from the ion at *m/z* 300.0274. In addition, the aglycone ion was prone to Retro-Diels Alder (RDA) cleavage, resulting in characteristic ions at *m/z* 151.0038 and 149.0249.

**Figure 3:**
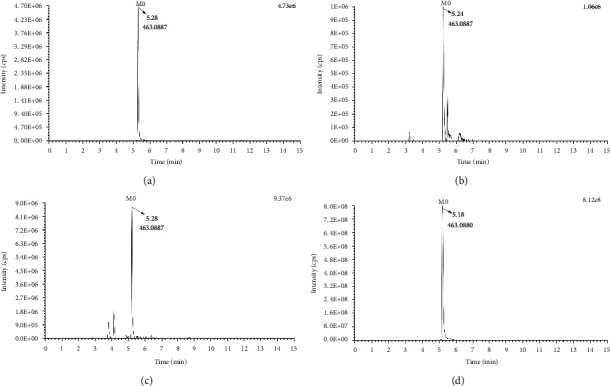
Extract ion chromatograms (EICs) of hyperoside: (a) Standard, (b) Plasma, (c) Urine and (d) feces.

The results showed that a total of 33 metabolites were identified, including 16 in plasma, 31 in urine, and 14 in feces. As shown in Figure 3, M0 was eluted at 5.2 min and exhibited [M-H]^−^ ion at *m/z* 463.08 (C_21_H_20_O_12_). It showed fragment ions at *m/z* 301.03, 300.02, 271.02, 255.02, 243.02, 227.03, 151.00, and 149.02 similar to hyperoside standard, indicating M0 was hyperoside and could be detected in rat plasma, urine, and feces. The identification information of each metabolite was shown in Table 1. Among them, M6, M8, M10, M14-15, M17-M23, and M27 were newly discovered metabolites in this study, which had not been reported in the identification literature up to now.

### 3.1. Metabolite Analysis in Plasma

**Figure 4:**
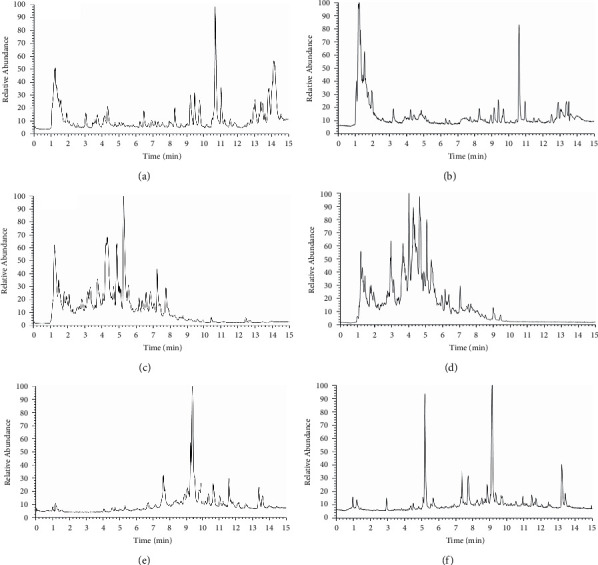
Total ion chromatograms (TICs) in rats: (a) Blank plasma, (b) Medicated plasma, (c) Blank urine, (d) Medicated urine, (e) Blank feces and (f) medicated feces.

**Figure 5:**
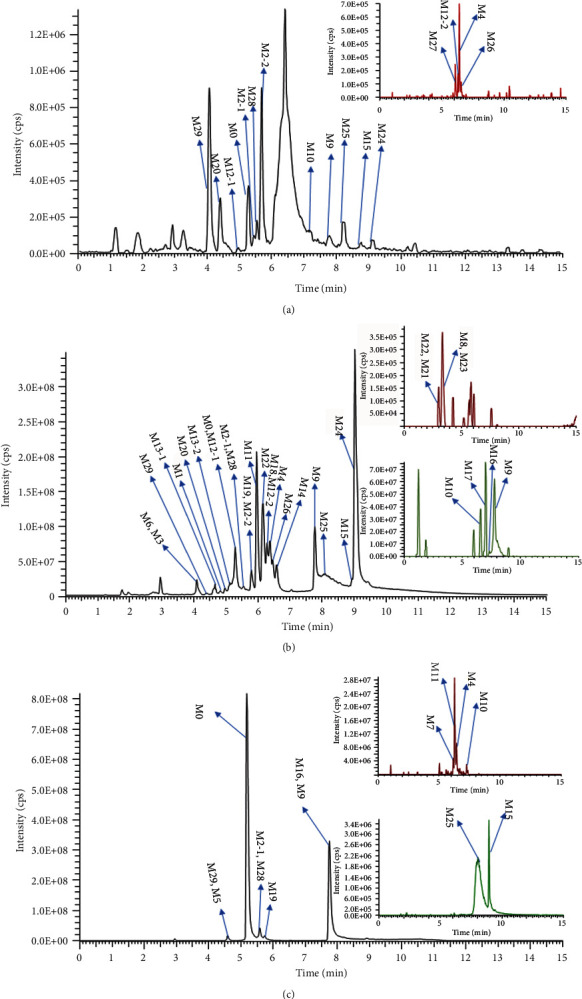
Extract ion chromatograms (EICs) of hyperoside and its metabolites in rats: (a) Plasma, (b) Urine and (c) feces.

A total of 16 metabolites including the prototype were identified in plasma compared with the blank control group. The total ion chromatogram (TIC) of blank plasma is shown in Figure 4(a) and hyperoside-contained plasma is shown in Figure 4(b). The extract ion chromatogram (EIC) of hyperoside and its metabolites in rat plasma is shown in Figure 5(a).

M2-1 and M2-2 were eluted at 5.52 and 5.84 min respectively. The [M-H]^−^ ion at *m/z* 477.10 was 14 Da (CH_2_) more than that of M0. So we conjectured M2 was a methylation product of M0, which was further confirmed by the fragment ions at *m/z* 315.08 ([M-H-C_6_H_10_O_5_]^−^) and 301.07 ([M-H-C_6_H_10_O_5_-CH_2_]^−^). The enzyme that catalyzes this reaction in mammals is catechol-O-methyltransferase (COMT), which methylates only one of the two adjacent catechol-hydroxyl groups [19]. According to the order of peaks, M2-1 was 4′-O-methyl-hyperoside and M2-2 was 3′-O-methyl-hyperoside respectively proved by literature [14].

M4 was eluted at 6.41 min. The formula of M4 was predicted as C_21_H_20_O_11_, an oxygen atom (16 Da) less than that of M0, indicating a hydroxyl group was removed from M0. Besides, M4 performed fragment ion at *m/z* 285.0403, also 16 Da less than that of M0 at *m/z* 301.0353. M4 had a similar fragmentation pattern to generate fragment ions at *m/z* 255.0298, 227.0350, and 151.0037 to M0, giving a further clue that M4 was a dehydroxylation product of M0.

**Figure 6:**
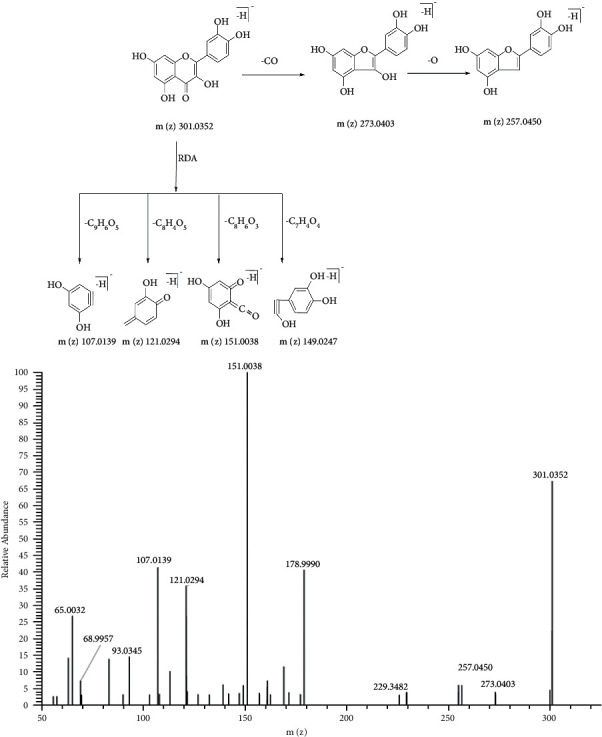
The fragment ion spectra and proposed fragmentation pathways of quercetin standard.

M9 was detected at 7.75 min. The [M-H]^−^ ion of M9 at *m/z* 301.0355 was 162 Da less than that of M0 suggesting a loss of galactoside (C_6_H_10_O_5_). The characteristic fragment ions at *m/z* 273.0406, and 257.0450 were formed after losing 28 Da (CO), and 16 Da (O) consecutively. And, other ions were at *m/z* 151.0038 and 149.0245 due to RDA cleavage from *m/z* 301.0355. So M9 might be quercetin, a product of deglycosylation of hyperoside. The fragment ion spectra and proposed fragmentation pathways of quercetin are shown in Figure 6.

M28 had a retention time of 5.54 min and generated the [M-H]^−^ ion at *m/z* 167.0348. It was speculated that the molecular formula was C_8_H_8_O_4_. Its MS^2^ spectrum displayed characteristic fragment ions at *m/z* 123.0452, 109.0217, and 93.0346 after the loss of CO_2_, CH_2_, and O. So M28 was considered to be 3,4-dihydroxyphenylacetic acid which was the decomposition product of M9 according to the literature [15].

### 3.2. Metabolite Analysis in Urine

31 compounds were detected in rat urine, of which 16 were also identified in plasma samples. Figures 4(c) and 4(d) show the TIC of blank urine and drug-contained urine respectively.  Figure 5(b) shows the EIC of hyperoside and its metabolites in rat urine.

M1 was detected at 4.95 min. In the MS^2^ spectrum, a peak of fragment ion at *m/z* 463.0877 was generated, which might be obtained from the [M-H]^−^ ion at *m/z* 639.1223 through removing a glucuronic acid (C_6_H_8_O_6_). And further fragment ions at *m/z* 271.0244, 255.0300, 227.0352, 151.0038, and 149.0244 were the symbols of hyperoside. It was speculated that M1 was the glucuronide conjugation product of hyperoside.

M11 showed the [M-H]^−^ ion at *m/z* 380.9928 with a retention time of 6.21 min. In the MS^2^ spectrum, a characteristic fragment ion at *m/z* 301.0354 was generated after losing 80 Da, indicating it was a product of sulfate conjugation of M9.

M13-1 and M13-2 were eluted at 4.63 and 5.15 min, respectively. The [M-H]^−^ ion was at *m/z* 653.09 and further produced fragment ions at *m/z* 477.06 ([M-H-C_6_H_8_O_6_]^−^) and 301.03 ([M-H-2C_6_H_8_O_6_]^−^), which suggested that they might be a diglucuronide conjugation product of M9.

M14 showed the [M-H]^−^ ion at *m/z* 557.0253 with a retention time of 6.63 min. M14 formed fragment ions at *m/z* 477.0679 and 301.0352 after the consecutive loss of 80 Da (SO_3_) and 176 Da (C_6_H_8_O_6_). M14 was presumed to be a sulfation and glucuronide conjugation product of M9.

M16 was eluted at 7.56 min and yielded the [M-H]^−^ ion at *m/z* 317.0313. The formula of M16 was predicted as C_15_H_10_O_8_, which added one oxygen atom (16 Da) to M9. The MS^2^ spectrum of M16 displayed characteristic fragment ions at *m/z* 289.1573 ([M-H-CO]^−^) and 257.0264 ([M-H-CO-2O]^−^), providing further evidence that M16 was a hydroxylation product of M9.

**Figure 7:**
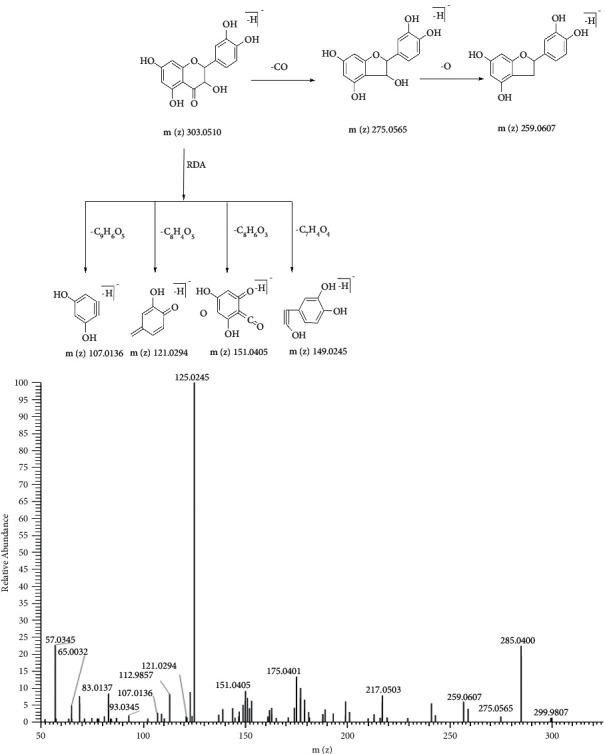
The fragment ion spectra and proposed fragmentation pathways of dihydroquercetin standard.

M19 was detected at 5.74 min. The [M-H]^–^ ion at *m/z* 303.0513 was 2 Da (2H) more than that of M9 at *m/z* 301.0355, indicating that the reduction reaction took place in the quercetin. This was also confirmed by the fragment ion at *m/z* 275.0442, 2 Da more than that of M9 at *m/z* 273.0406. Therefore, M19 was considered to be dihydroquercetin, a hydrogenation product of M9. The fragment ion spectra and proposed fragmentation pathways of dihydroquercetin are shown in Figure 7.

### 3.3. Metabolite Analysis in Feces

14 compounds were annotated in rat feces, and only 2 compounds were not identified in rat plasma and urine. Figures 4(e) and 4(f) show the TIC of blank feces and drug-contained feces respectively.  Figure 5(c) shows the EIC of hyperoside and its metabolites in rat feces.

M5 had a retention time of 4.53 min. The [M-H]^−^ ion at *m/z* 479.0834 was 16 Da (O) more than that of M0, indicating a hydroxyl group was added to M0. The fragment ions at *m/z* 317.0283 and 301.0251 were generated after the consecutive loss of galactoside (C_6_H_10_O_5_) and an oxygen atom (O). So M5 was predicted as a hydroxyl product of M0.

M7 was detected at 6.19 min and yielded the [M-H]^−^ ion at *m/z* 505.0990, 42 Da (C_2_H_2_O) more than that of M0, indicating that acetyl might be attached. Then, M7 directly formed fragment ions at *m/z* 301.0277, 271.0247, and 243.0298, giving a further clue that it was an acetylation product of M0. Therefore, M7 was probably acetyl-hyperoside.

In the results of this experiment, M6, M8, M10, M14-15, M17-M23, and M27 were hyperoside metabolites that had not been reported previously, and these metabolites might also have other biological activities [20]. For example, hyperoside was hydrolyzed to form its aglycone quercetin, as an intermediate metabolite, quercetin continued to undergo hydro reduction reaction to produce dihydroquercetin (M19).

Dihydroquercetin, also known as taxifolin, has anticancer, antibacterial, scavenging free radicals, and other effects [21] but has a low content in plants [22]. And, this metabolic pathway had not been reported in the literature on hyperoside or quercetin metabolism *in vivo*. Therefore, new research contents were added to this study. In addition, dihydroquercetin is a flavonoid compound with high antioxidant, capillary-protective, and anti-inflammatory activity [23]. Some studies have shown that dihydroquercetin was more effective than quercetin in inhibiting the superoxide produced by xanthine oxidase and has a stronger antioxidant effect [24, 25]. There were also studies that showed dihydroquercetin pretreatment markedly alleviated cardiac dysfunction, scavenged free radicals, reduced lipid peroxidation, and increased the activity of antioxidant enzymes *ex vivo* and *in vitro* [26]. So it was proposed to be useful in the prevention and treatment of cardiovascular disease.

### 3.4. Proposed Metabolic Pathways of Hyperoside in Rats

**Figure 8:**
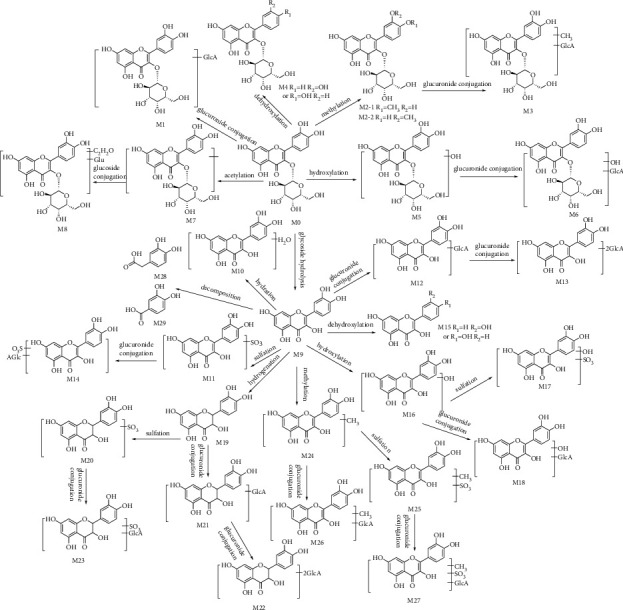
The proposed metabolic pathways of hyperoside in rats.

In this experiment, UPLC-Q-Exactive Orbitrap MS was used to systematically identify hyperoside and its metabolites in rat plasma, urine, and feces, so as to clarify the metabolic pathways of hyperoside *in vivo*. A total of 33 metabolites were identified, including 10 phase I metabolites and 23 phase II metabolites. The metabolic pathways of hyperoside in rats included phase I metabolic reactions such as glycoside hydrolysis, hydroxylation, dehydroxylation, hydrogenation, and hydration, and phase II metabolic reactions such as methylation, acetylation, glucuronidation, and sulfation. The analysis results showed that the type of phase II metabolites detected in urine was the most abundant, indicating that hyperoside was easy to combine with some endogenous substances such as glucuronide and sulfate after entering the body, resulting in the increasing of polarity and water solubility of the metabolites, which were more likely to be excreted through the urine [27]. The nine metabolic reactions of hyperoside involved and the number of metabolites in rat plasma, urine, and feces are shown in Table 2 (duplicate metabolites in diverse samples were only counted at once). The proposed metabolic pathways of hyperoside in rats are shown in Figure 8.

In total, 10 metabolites were produced by phase I metabolism. Hydroxylation, dehydroxylation, glucoside hydrolysis, hydrogenation, and hydration were the main first-stage metabolic pathways. Hyperoside lost the hydroxyl group to produce M4 and reacted with the hydroxyl group to generate M5, respectively. Glycoside hydrolysis occurred to produce M9, and aglycone M9 was used as an intermediate metabolite for further metabolic transformations *in vivo*. M9 hydrogenation produced M19. M10 was formed by hydration, M15 and M16 by dehydroxylation and hydroxylation, and M28 and M29 by cleavage of M9.

The other 23 metabolites were produced by phase II metabolism. Methylation, acetylation, sulfation, and glucuronic acid combination were the main second-stage metabolic pathways. Hyperoside was replaced by methyl to form M2 and acetyl to form M7 and M8. M24 was formed by M9 methylation reaction. Methylation and acetylation reactions relatively reduce the polarity of metabolites, making them easier to be absorbed by tissues through biofilms and preventing drug removal [28]. Hyperoside and some of its phase I metabolites further reacted with a glucuronic acid group to form M1, M3, M6, M12, M13, M18, M21, M22, and M26 and sulfate group to form M11, M17, M20, and M25. Besides, M14, M23, and M27 were formed through the complex reactions of sulfation and glucuronidation. Compared with hyperoside, the polarity and water solubility of these metabolites increased, which was conducive to excretion.

## 4. Conclusion

In this study, UPLC-Q-Exactive Orbitrap MS was used to establish an accurate and systematic analysis method to study the biotransformation of hyperoside in rats. The fragmentation patterns of hyperoside in rats were summarized. Hyperoside was prone to glycoside hydrolysis *in vivo*, resulting in producing aglycone quercetin. Quercetin, as an intermediate product, further went through other metabolic transformations *in vivo*. The neutral loss of (CO + OH), COH, CO, O, and RDA cleavage were also used as the specific fragmentation rules of this compound, which provided the basis for rapid screening of hyperoside metabolites. Thus, a total of 33 metabolites were found in rats, including 16 in plasma, 31 in urine, and 14 in feces, of which 10 were phase I metabolites and 23 were phase II metabolites. Besides, the metabolites quercetin and dihydroquercetin were unambiguously confirmed by comparison with standard substances. The metabolic pathways of hyperoside in rats included hydrolysis, hydroxylation, dehydroxylation, hydro reduction, hydration, methylation, acetylation, glucuronidation, and sulfation. In this study, 13 metabolites had not been reported in the articles related to hyperoside or quercetin metabolism *in vivo*, and these metabolites might also have many potential drug activities.

## Figures and Tables

**Table 1:** Characterization of hyperoside and its metabolites in rats using UPLC-Q-exactive orbitrap MS.

No.	Formula	Calculated (*m/z*)	Observed (*m/z*)	Error (ppm)	*t*_R_ (min)	Major fragments (*m/z*)	Metabolic reactions	P	U	F
M0	C_21_H_20_O_12_	463.0871	463.0887	3.46	5.28	301.0353, 300.0276, 271.0247, 255.0297, 243.0299, 227.0351, 151.0039, 149.0249	Parent	+	+	+

M1	C_27_H_28_O_18_	639.1192	639.1223	4.85	4.95	463.0877, 301.0352, 271.0244, 255.0300, 243.0297, 227.0352, 151.0038, 149.0244	Glucuronide conjugation	−	+	−

M2-1	C_22_H_22_O_12_	477.1028	477.1047	3.98	5.52	315.0881, 301.0713, 255.0659, 227.0342, 151.0038,149.0249	Methylation	+	+	+

M2-2	C_22_H_22_O_12_	477.1028	477.1042	2.93	5.84	315.0878, 301.0355, 255.0301, 243.0292, 227.0353, 151.0038, 149.0246	Methylation	+	+	−

M3	C_28_H_30_O_18_	653.1348	653.1375	4.13	4.07	477.1042, 315.0511, 301.0356, 271.0247, 255.0299, 243.0301, 227.0350, 151.0037	Methylation, glucuronide	−	+	−

M4	C_21_H_20_O_11_	447.0922	447.0939	3.80	6.41	285.0403, 255.0298, 227.0350, 151.0037, 149.0611	Dehydroxylation	+	+	+

M5	C_21_H_20_O_13_	479.082	479.0834	2.92	4.53	317.0283, 301.0251, 271.0244, 243.0283, 227.0348, 151.0039	Hydroxylation	−	−	+

M6	C_27_H_28_O_19_	655.1141	655.1157	2.44	4.03	479.1103, 317.0567, 301.0316, 271.0246, 255.0295, 243.0300, 227.0348, 151.0039	Hydroxylation, glucuronide	−	+	−

M7	C_23_H_22_O_13_	505.0977	505.099	2.57	6.19	301.0277, 271.0247, 255.0298, 243.0298, 227.0357, 151.0042	Acetylation	−	−	+

M8	C_29_H_32_O_18_	667.1504	667.1555	7.64	3.53	301.0276, 271.0247, 255.0298, 243.0299, 227.0351, 151.0038,149.9955	Acetylation, glucoside	−	+	−

M9	C_15_H_10_O_7_	301.0343	301.0355	3.99	7.75	273.0406, 257.0450, 151.0038, 149.0245, 121.0295, 107.0139, 65.0032	Glycoside hydrolysis	+	+	+

M10	C_15_H_12_O_8_	319.0448	319.0458	3.13	7.02	301.0328, 273.0894, 151.9274, 149.0975, 121.0296, 107.0503, 65.0396	Glycoside hydrolysis, hydration	+	+	+

M11	C_15_H_10_O_10_S	380.9911	380.9928	4.46	6.21	301.0354, 273.0407, 151.0038, 149.0248, 121.0296, 107.0139, 65.0033	Glycoside hydrolysis, sulfation	−	+	+

M12-1	C_21_H_18_O_13_	477.0664	477.0678	2.93	5.26	301.0354, 273.0392, 257.0462, 151.0038, 149.0244, 121.0296, 107.0139, 65.0032	Glycoside hydrolysis, glucuronide	+	+	−

M12-2	C_21_H_18_O_13_	477.0664	477.0678	2.93	6.36	301.0355, 273.0401, 257.0460, 151.0038, 149.0243, 121.0296, 107.0139, 65.0033	Glycoside hydrolysis, glucuronide	+	+	−

M13-1	C_27_H_26_O_19_	653.0985	653.1015	4.59	4.63	477.0639, 301.0356, 273.0403, 151.0038, 149.0243, 121.0296, 107.0140, 65.0032	Glycoside hydrolysis, glucuronide × 2	−	+	−

M13-2	C_27_H_26_O_19_	653.0985	653.1014	4.44	5.15	477.0671, 301.0353, 273.0392, 257.0452, 151.0038, 149.0244, 121.0296, 107.0139, 65.0033	Glycoside hydrolysis, glucuronide × 2	−	+	−

M14	C_21_H_18_O_16_S	557.0232	557.0253	3.77	6.63	477.0679, 380.9921, 301.0352, 273.0405, 151.0037, 149.0243, 121.02965, 107.0139, 65.0032	Glycoside hydrolysis, sulfation, glucuronide	−	+	−

M15	C_15_H_10_O_6_	285.0394	285.0406	4.21	8.92	257.0453, 241.0500, 151.0038, 121.0295, 65.0033	Glycoside hydrolysis, dehydroxylation	+	+	+

M16	C_15_H_10_O_8_	317.0292	317.0313	6.62	7.56	289.1573, 257.0264, 151.1128, 149.1339, 121.0296, 65.3057	Glycoside hydrolysis, hydroxylation	−	+	+

M17	C_15_H_10_O_11_S	396.986	396.9881	5.29	7.15	317.0305, 289.1569, 151.0039, 149.1339, 121.0296, 65.4439	Glycoside hydrolysis, hydroxylation, sulfation	−	+	−

M18	C_21_H_18_O_14_	493.0613	493.0627	2.84	6.34	317.0573, 289.0638, 273.0349, 151.0039, 149.9958	Glycoside hydrolysis, hydroxylation, glucuronide	−	+	−

M19	C_15_H_12_O_7_	303.0499	303.0513	4.62	5.74	275.0442, 259.1820, 151.0402, 149.0245, 121.0295, 107.0139	Glycoside hydrolysis, hydrogenation	−	+	+

M20	C_15_H_12_O_10_S	383.0067	383.0079	3.13	5.06	303.0514, 285.0403, 275.1660, 151.0036, 123.0691, 109.0533,	Glycoside hydrolysis, hydrogenation, sulfation	+	+	−

M21	C_21_H_20_O_13_	479.082	479.0834	2.92	3.42	303.0873, 285.0763, 275.0457, 257.0373, 151.0098, 123.0455, 109.0295	Glycoside hydrolysis, hydrogenation, glucuronide	−	+	−

M22	C_27_H_28_O_19_	655.1141	655.1157	2.44	3.38	479.1103, 303.0405, 151.0039	Glycoside hydrolysis, hydrogenation, glucuronide × 2	−	+	−

M23	C_21_H_20_O_16_S	559.0388	559.04	2.15	3.54	479.0675, 303.0414, 151.0038, 109.0295	Glycoside hydrolysis, hydrogenation, sulfation, glucuronide	−	+	−

M24	C_16_H_12_O_7_	315.0499	315.0511	3.81	9	300.0275, 283.0241, 271.0246, 255.0296, 243.0299, 227.0354, 151.0038, 149.9958, 121.6068, 107.0139,	Glycoside hydrolysis, methylation	+	+	−

M25	C_16_H_12_O_10_S	395.0067	395.0084	4.30	8.02	315.0511, 300.0277, 283.0244, 271.0247, 255.0306, 243.0300, 227.0345, 151.0038, 149.9956, 121.6540, 107.0139	Glycoside hydrolysis, methylation, sulfation	+	+	+

M26	C_22_H_20_O_13_	491.082	491.0833	2.65	6.56	315.0511, 300.0276, 283.0251, 271.0247, 255.0300, 243.0297, 227.0353, 151.0038, 149.9959, 121.6044, 107.0139	Glycoside hydrolysis, methylation, glucuronide	+	+	−

M27	C_22_H_20_O_16_S	571.0388	571.0408	3.50	6.26	315.0511, 300.0278, 271.0247, 255.0299, 243.0292, 227.0346, 151.0037, 149.9953, 107.0139	Glycoside hydrolysis, methylation, sulfation, glucuronide	+	+	−

M28	C_8_H_8_O_4_	167.0339	167.0348	5.39	5.54	123.0452, 109.0217, 93.0346	Decomposition	+	+	+

M29	C_7_H_6_O_4_	153.0182	153.0191	5.88	4.5	109.0659, 93.0346	Decomposition	+	+	+

*Notes*. P represents plasma samples, U represents urine samples and F refers to feces samples.

**Table 2:** Metabolic reactions of hyperoside in rat plasma, urine and feces.

Metabolic type	Reaction type	Changes in the group	Number of metabolites
Phase I metabolism	Glycoside hydrolysis	−C_6_H_10_O_5_	21
	Hydroxylation	+O	5
	Dehydroxylation	−O	2
	Hydrogenation	+H_2_	5
	Hydration	+H_2_O	1

Phase II metabolism	Methylation	+CH_2_	7
	Acetylation	+C_2_H_2_O	2
	Glucuronide conjugation	+C_6_H_8_O_6_	14
	Sulfation	+SO_3_	7

## Data Availability

The data used to support the findings of this study are included within the manuscript and are available from the corresponding author upon request.
